# Gut microbiota in atherosclerosis: focus on trimethylamine N‐oxide

**DOI:** 10.1111/apm.13038

**Published:** 2020-03-30

**Authors:** Yingqian Zhu, Qingqing Li, Hua Jiang

**Affiliations:** ^1^ Department of Geriatrics Shanghai East Hospital Tongji University School of Medicine Shanghai China

**Keywords:** Atherosclerosis, cardiovascular disease, gut microbiota, microbial metabolites, trimethylamine N‐oxide

## Abstract

The increasing prevalence of cardiovascular diseases cannot adequately be explained by traditional risk factors. Recently, accumulating evidence has suggested that gut microbiota‐derived numerous metabolites are contributors to atherosclerotic events. Among them, the role of trimethylamine N‐oxide (TMAO) in promoting atherosclerosis has gained attention. TMAO is reported to exert the proatherogenic effects by impacting on the traditional risk factors of atherosclerosis and is associated with high risk of cardiovascular events. Besides that, TMAO is involved in the complex pathological processes of atherosclerotic lesion formation, such as endothelial dysfunction, platelet activation and thrombus generation. In light of these promising findings, TMAO may serve as a potential target for atherosclerosis prevention and treatment, which is conceptually novel, when compared with existing traditional treatments. It is likely that regulating TMAO production and associated gut microbiota may become a promising strategy for the anti‐atherosclerosis therapy.

Atherosclerosis, a chronic disease of arteries and prime culprit of cardiovascular diseases, has been a leading cause of mortality worldwide for many years (1). A broad spectrum of traditional cardiovascular risk factors associated with atherosclerosis includes hypertension, hyperlipidemia, diabetes mellitus, obesity and smoking (2, 3). These factors to some extent explain the pathogenesis of atherosclerosis, but are not completely implicated in cardiovascular diseases (4). At the presents, anti‐atherosclerosis therapies targeting the traditional risk factors include first‐line antiplatelet drugs, hypoglycemic drugs and lipid‐lowering agents. Despite the standard and efficacious conventional therapies, cardiovascular diseases risk still persists in patients (5, 6), which may be partially attributed to complex etiology of atherosclerosis. There is a pressing need to improve our understanding of atherosclerosis and develop effective approaches targeting underlying mechanisms.

Recent researches have highlighted the importance role of gut microbiota in human health (7). Gut microbiota is an ecological community of microorganisms inhabiting along the gastrointestinal tract, which consists of trillions of bacteria and encodes more than 100 times as many genes as human genome (8). Gut microbiota metabolizes some dietary nutrients (e.g. phosphatidylcholine, choline, carnitine) as substrates and eventually generates trimethylamine N‐oxide (TMAO), which is a bioactive gut microbiota‐derived metabolite (9). Emerging evidence has indicated TMAO may be involved in the development of atherosclerosis, and the interaction between TMAO and atherosclerosis has gained attention (10, 11, 12, 13).

Trimethylamine N‐oxide (TMAO) was initially recognized as a product waste without action, but nowadays there is a lot convincing evidence showing that TMAO participates in a variety of biological reactions actively and affects activities of enzymes and hormones within human body (14). In particular, it has been suggested that TMAO played an important role in pathophysiology of atherosclerosis (15, 16). Some preclinical studies have found that consumption of TMAO could lead to atherosclerotic lesion development (13, 15, 17, 18). Cohort studies showed that the increasing plasma TMAO levels were correlated with high cardiovascular risk (19, 20, 21, 22, 23, 24). However, the specific mechanism is complex and not fully elucidated. In this review, we will focus on the metabolism of TMAO, how TMAO leads to development of atherosclerosis and the recent advances in our understanding of potential therapeutic strategies for atherosclerosis and cardiovascular diseases. The present review in our knowledge may provide a basis for the improvement of anti‐atherosclerosis therapy in the future.

## PRODUCTION AND METABOLISM OF TMAO

**Fig. 1:**
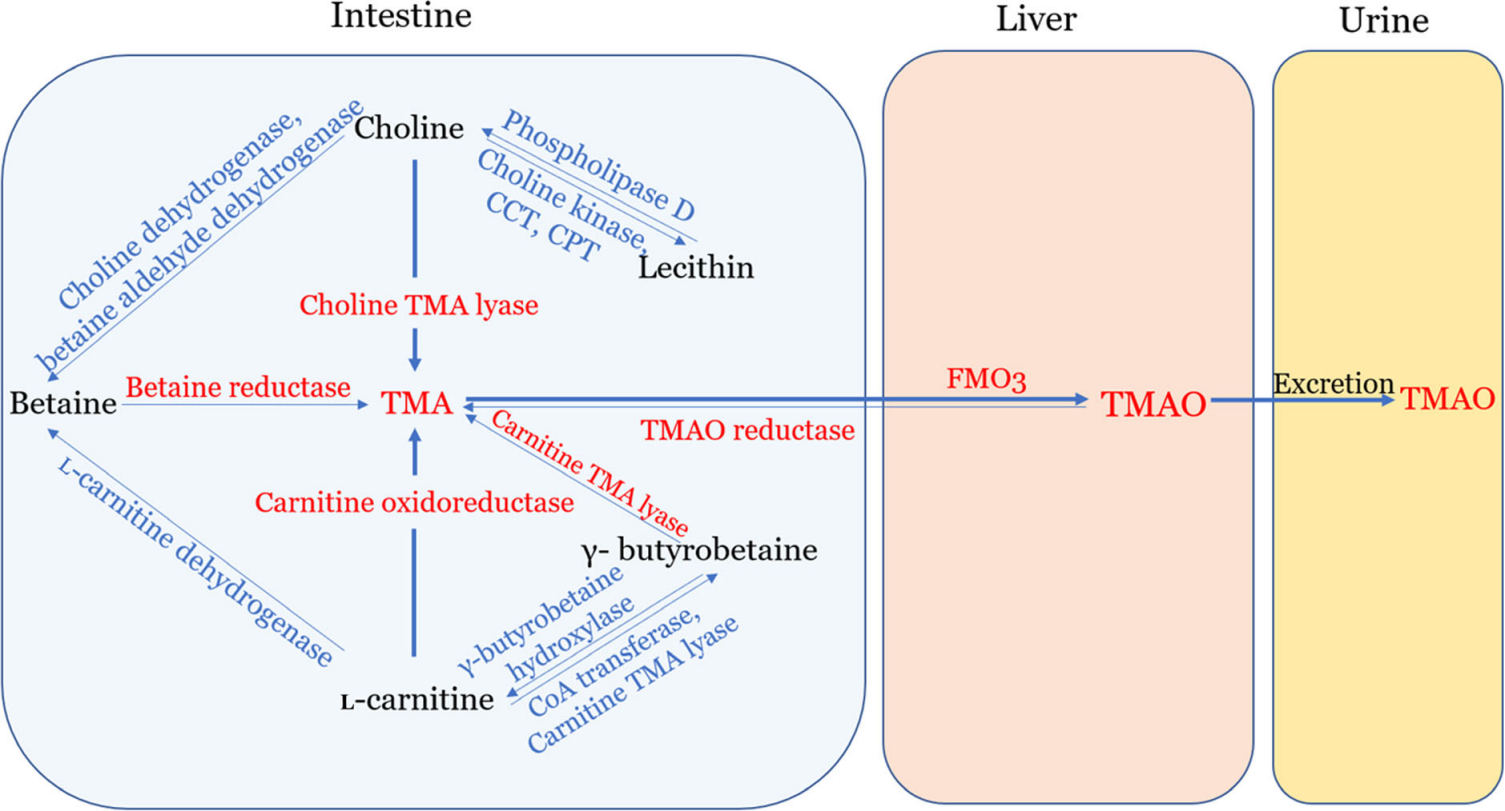
Production and metabolism of TMAO. Trimethylamine (TMA) is generated from dietary choline and L‐carnitine in the intestinal tract by the action of multiple enzymes. Then, it is absorbed from intestinal enterocyte membranes and delivered to the liver, where TMA is rapidly further oxidized by flavin monooxygenase 3（FMO3）to form trimethylamine N‐oxide （TMAO）. Afterward, the majority of TMAO is excreted in urine. CCT: phosphocholine cytidylyltransferase; CPT: choline phosphotransferase.

Trimethylamine N‐oxide (TMAO) is an amine oxide mainly derived from the oxidation of trimethylamine (TMA), the intermediate product of the microbial metabolic pathway (15). TMA can be primarily generated from dietary choline and L‐carnitine by intestinal bacteria and absorbed into hepatic portal circulating blood (25). TMA is further oxidized by hepatic enzymes flavin monooxygenases 3(FMO3) in the host liver and generates TMAO, which is the end product and cannot be metabolized further (17). The majority of TMAO can be excreted unchanged in the urine by the kidney within 24 h (26). The remaining TMAO is reduced to TMA by the action of TMAO reductase (27) (Fig. 1).

Dietary choline and L‐carnitine are two principle nutrients precursors of TMAO, which are metabolized to TMA by gut microbiota and various enzymes. Regarding to choline, free choline and choline esters, such as phosphatidylcholine (PC), also known as lecithin, can be found in high quantities in many foodstuffs, for example, egg yolks, liver, meats, high‐fat dairy products, as well as some certain nuts and beans (28, 29). As an essential nutrient for human, choline is the precursor of the phospholipids required for maintaining cell membranes and supporting cholinergic neurotransmission (30). Choline can be ingested and directly converted into TMA by choline TMA lyase (25). Choline is oxidized to betaine by the collaborative action of two enzymes, the choline dehydrogenase and betaine aldehyde dehydrogenase (31). As a common ingredient in many plants, betaine can be transformed into TMA further, catalyzed by betaine reductase. In addition to betaine, lecithin is another important metabolite of choline, which is synthesized by the catalytic action of three enzymes sequentially: choline kinase, phosphocholine cytidylyltransferase (CCT) and choline phosphotransferase (CPT) (32). In turn, lecithin can be reconverted into choline again catalyzed by phospholipase D (29) (Fig. 1).

L‐carnitine is another dietary precursor of TMAO fond predominantly in red meat and dairy products. As an essential nutrient in humans, L‐carnitine is mainly responsible for the fatty acid transportation. The production of TMA from L‐carnitine appears to be either direct or indirect (31, 33). Carnitine oxidoreductase is the main enzyme that transforms L‐carnitine into TMA directly (33). Alternatively, L‐carnitine can be converted to TMA indirectly via generating γ‐butyrobetaine firstly. This reaction is catalyzed by either two kinds of key enzymes, the carnitine CoA transferase and/or carnitine TMA lyase. There is bidirectional conversion between γ‐butyrobetaine and L‐carnitine with γ‐butyrobetaine being catalyzed by γ‐butyrobetaine hydroxylase. Then, γ‐butyrobetaine can be converted into TMA by the action of carnitine TMA lyase (31, 34, 35). Betaine is another TMA precursor, which can be catalyzed by L‐carnitine dehydrogenase and then reduced to TMA by betaine reductase (Fig. 1).

The circulating levels of TMAO are positively associated with the consumption of its metabolic precursors, which are derived from dietary sources, including meat, eggs, dairy products and salt water fish. The amounts of TMAO production are determined not only by diet preferences, but to a large extent also by microbial composition (15, 36). As the major nutrient precursor for TMAO, choline can be decomposed by an array of biological reactions involving the splitting of the carbon–nitrogen bond of choline. These processes are regulated by gut microbiota, particularly by the phylum *Firmicutes,* phylum *Proteobacteria* and six microbial genera, such as *Anaerococcus hydrogenalis, Clostridium asparagiforme, Clostridium hathewayi, Clostridium sporogenes, Escherichia fergusonii, Proteus penneri, Providencia rettgeri* and *Edwardsiella tarda* (37, 38). Also, gut microbiota plays a crucial role in the metabolism of L‐carnitine by cleaving the 3‐hydroperoxybutyryl moiety. The main microbial species responsible for the degradation of L‐carnitine are *proteobacteria and Bacteroidetes* at phyla level and *Prevotellaceae* at family level (15, 33, 39). More notably, in line with the obligatory role of gut microbiota played in TMAO production, studies have shown that the use of broad‐spectrum antibiotics altered gut microbiota composition and reduced TMAO levels, which indicated the importance of gut microbiota in the metabolism of TMAO (15).

## THE TMAO AND ATHEROSCLEROSIS

Recent clinical studies have claimed the close associations of TMAO with atherosclerotic events. In a large cohort of healthy participants (N = 4007), the elevated levels of TMAO were positively correlated with the increasing incidence of major adverse cardiovascular events (including stroke, myocardial infarction and death) after 3 years of follow‐up (20). High levels of plasma TMAO also presented incremental prognostic value for adverse outcomes and all‐cause mortality. For atherosclerotic patients with peripheral artery disease (PAD), TMAO levels were associated with a 2.7‐fold risk of mortality independently (23). In a cohort study of patients with acute coronary syndromes (ACS), the levels of TMAO indicated long‐term (7‐year) and short‐term (30 days and 6 months) mortality (24). Intriguingly, even for the patients with stable ACS received the optimal treatments, the elevated levels of TMAO were also found to be predictors for long‐term mortality risk (40). Further, TMAO also portended adverse outcomes in atherosclerotic patients when combined with other chronic diseases, including heart failure, chronic kidney disease, stroke, acute myocardial infarction and diabetes mellitus (41, 42, 43, 44, 45, 46). A dose–response meta‐analysis indicated that each 10 μmol/L increment of TMAO corresponded to a 7.6% increase in the risk of all‐cause mortality (47).

Carotid intima‐media thickness (C‐IMT) is a non‐invasive ultrasound index for the common carotid, serving as the surrogate marker and indicator of early atherosclerosis (48). It was associated with high cardiovascular risk and greatly elevated in patients with coronary artery disease, which predicted the severity and complexity of clinical coronary events in the general population (49, 50). In an epidemiological study, C‐IMT was positively correlated with circulating TMAO levels, independently of the strong determinants of cardiovascular diseases, including age, sex and visceral fat mass (22). However, controversy exists concerning the role of TMAO in early atherosclerosis due to few relevant data available. In a case–control study, patients with carotid artery atherosclerosis did not present increased serum levels of TMAO compared with corresponding healthy participants, but the levels of TMAO precursor, L‐carnitine were detected increased in atherosclerotic patients (51). Another population‐based study showed that TMAO was related to C‐IMT but did not promote the development of early atherosclerosis (52). Intriguingly, the inconsistent results can be explained when taking into considerations of age distribution in the study, since all the participants were from 33 to 45 years of age. The age range had a much lower risk burden in the sample population. Moreover, after lifestyle intervention, the C‐IMT thickness significantly decreased in subjects who had the largest decline of TMAO concentrations (22).

## TMAO IN ATHEROSCLEROSIS

**Fig. 2:**
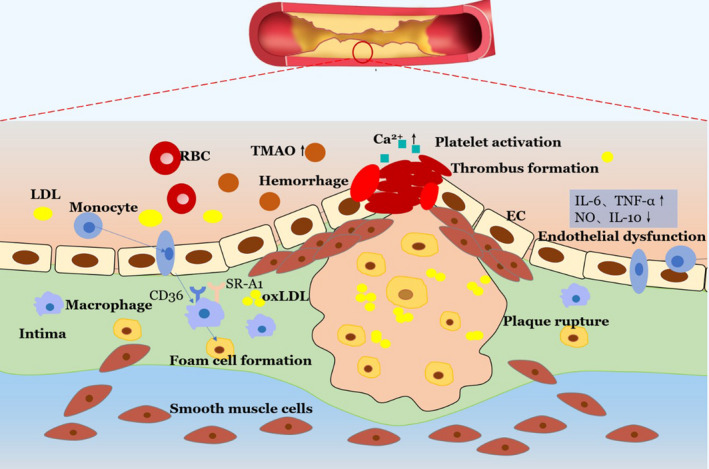
The role of TMAO in atherosclerotic lesion formation and development. The high levels of TMAO in circulation have a crucial role in the foam cell formation and endothelial dysfunction; TMAO can activate platelet and promote thrombus generation, making the atherosclerotic plaque vulnerable to rupture.

Emerging evidence has demonstrated the association of high circulating TMAO levels with increased cardiovascular diseases risk (9, 20). TMAO is a proatherogenic factor, which is actively involved in atherosclerosis development and progression (9, 15). Even the size of aortic atherosclerotic plaque is positively correlated with the plasma levels of TMAO (9). TMAO plays a critical role in the atherosclerotic lesion formation and development, and the pathogenic mechanisms are highlighted in Fig. 2.

## FOAM CELLS FORMATION

The accumulation of foam cells in arterial intima is the cellular hallmark and earliest step of atherogenesis. Foam cells exert a proatherogenic effect from the initial lesion formation to plaques rupture. The majority of foam cells originate from macrophages which can regulate lipoprotein metabolism. It seems that TMAO participates in the pathological process of atherosclerosis development by promoting macrophage migration as well as transformation of macrophage into foam cell (9, 15). In atherosclerosis, low‐density lipoproteins (LDLs) in the vascular walls tend to be oxidatively modified. After differentiation from monocytes initially, macrophages can sense and intake the oxidized modified LDLs (ox‐LDL) by the action of cluster of differentiation 36(CD36) and class A1 scavenger receptors (SR‐A1), which are the macrophage scavenger receptors (SRs) with high affinity to sense ox‐LDL (53, 54). TMAO plays an important role in augmenting the accumulation of ox‐LDL within macrophages by upregulating the expression of CD36 and SR‐A1 (55, 56). In this process, more macrophage cells are transformed into foam cells. Besides that, TMAO also promotes macrophage migration and increases the expression of inflammatory cytokines, including TNF‐α and IL‐6. When responding to inflammatory activation, these macrophages can penetrate through endothelial barrier and accumulate in vessel intima, contributing to the atherosclerotic plaque formation (53).

## ENDOTHELIAL DYSFUNCTION

Endothelial dysfunction is the key event in the development of atherosclerotic lesions and cardiovascular diseases. Growing set of evidence has indicated that gut microbiota‐derived TMAO and its dietary precursors are involved in endothelial dysfunction and atherosclerosis (9, 57). Mice fed a choline‐rich diet exhibited obvious dyslipidemia and hyperglycemia with impaired damage of vascular endothelium (58). A clinical study first demonstrated that an elevated plasma level of TMAO was significantly associated with impaired endothelial function, increased inflammation and fewer circulating endothelial progenitor cells (EPCs) in patients with stable angina (59). The study then confirmed that high‐TMAO levels inhibited circulating EPCs via increasing intracellular inflammation and oxidative stress, eventually leading to endothelial dysfunction (59). TMAO can aggravate inflammation via the signaling of mitogen‐activated protein kinase and nuclear factor‐κB (60). TMAO also downregulates the expression of anti‐inflammatory cytokine IL‐10, which can protect endothelium cells from damage from increased inflammation and oxidative stress (61). In addition, TMAO significantly aggravates the responses of oxidative stress, leading to generation of reactive oxygen species (ROS) and reduction of nitric oxide (NO), which exerts negative effects in maintaining the normal vascular function (61, 62). Furthermore, TMAO damages the self‐repairing capacity of injured endothelial cells (63). All of the pathophysiological processes that TMAO involved in eventually lead to endothelial dysfunction and atherosclerosis.

## PLAQUE INSTABILITY

Atherosclerotic plaques are composed of a lipid‐rich atheromatous core, covered by the collagen‐rich fibrous cap. The accumulating of the lipid atheromatous component weakens the fibrous cap and enhances inflammation, leaving the plaque vulnerable to rupture (64). If it ruptured, the thrombogenic interior arterial wall is exposed and susceptible to thrombus formation, which leads to serious cardiovascular events, such as acute coronary syndrome and stroke (65, 66). Considering the role of TMAO in pathogenesis of atherosclerosis, several lines of evidence have explored the relationship between plasm TMAO levels and the instability of coronary plaque. In a cohort study of patients with coronary artery disease (CAD), the results suggested that TMAO levels in the plaque rupture group were dramatically higher than those in nonplaque rupture group (67). The study further divided all patients into a thin‐cap fibroatheroma (TCFA) group and a non‐TCFA group according to the definition of TCFA (a plaque with lipid component in ≥ 2 quadrants with the thinnest thickness of fibrous cap ≤ 65 μm). There was a consistent tendency toward increasing TMAO levels and high incidence of TCFA (67). A subsequent study with 90 CAD patients further confirmed the previous data. All subjects were divided into a high‐TMAO group and a low‐TMAO group, and plaques in the high‐TMAO group exhibited lower fibrous cap thickness and higher incidence of TCFA. TMAO levels were significantly associated with the high prevalence of TCFA, with relatively high specificity and sensitivity in predicting the incidence of TCFA (68).

Although current evidence has suggested that increased plasm TMAO levels can result in vulnerability and even rupture of atherosclerotic plaques, the underlying mechanism is still lacking. It is possible that TMAO participates in the formation of microvessels within a coronary plaque, which leads to the imbalance between antiangiogenic and proangiogenic factors (69). The microvessels would become immature, leaving the plaque more vulnerable to rupture. Nevertheless, at present there is little convincing evidence to support the notion and the underlying mechanisms remain to be illuminated.

## PLATELET ACTIVATION AND THROMBUS GENERATION

Platelet activation, aggregation and artery occlusion are essential thrombogenic processes. High platelet reactivity is associated with enhanced potential of thrombotic events, which plays a pivotal role in subsequent cardiovascular events. As important contributor to atherogenic process, platelets activation is involved in thrombus development and rupture of atherosclerotic plaques, which in turn promotes platelets activation and aggravates atherosclerosis. A recent population‐based study showed that TMAO enhanced platelet hyperreactivity, independently predicting high risk of thrombosis (70). The study also revealed a dose‐dependent association between TMAO levels and platelets activation (71). Some preclinical studies have given valuable insights on pathogenic effects of TMAO on platelets function involved in atherosclerosis, although the precise mechanism remains more understood. It is suggested that intracellular cytosolic calcium [Ca^2+^]_i_ is the precursor to thrombus formation (72); intriguingly, TMAO can promote stimulus‐dependent [Ca^2+^]_i_ mobilization. Exposure to circulating TMAO triggers more [Ca^2+^]_i_ released from intracellular Ca^2+^ stores, which leads to platelet aggregation (70). High circulating levels of TMAO also strengthen platelet adhesion to immobilized collagen within whole blood, which substantially promotes platelet responsiveness to several more agonists and induces the cascade reaction of thrombus generation.

## THE INFLUENCE OF TMAO ON RISK FACTORS OF ATHEROSCLEROSIS

### Hypertension

Hypertension is the global public health concern and key risk factor for cardiovascular events. In recent years, the existence of links between dysbiosis of gut microbiota, microbial metabolites and blood pressure (BP) has been illuminated based on preclinical findings. In the rodent model of hypertension, hypertensive rats exhibited altered gut microbial compositions with decreased richness and diversity, which were sequenced precisely via high‐throughput pyrosequencing approach (73). It was suggested that gut microbiota promoted hypertension progression via regulating BP associated hormones, such as serotonin, dopamine and norepinephrine (74). Animal study showed that TMAO only enhanced BP of hypertensive animals, but cannot affect BP of normotensive animals. It was explained that TMAO exerted an indirect effect on hypertension via prolonging the hypertensive effect of Ang II, an important hormone in hemodynamic regulation (75). Furthermore, hypertension impairs gut–blood barrier and increases intestinal permeability of TMAO to blood circulation, making the situation worse (76). These findings may strengthen the positive correlation between elevated plasm levels of TMAO and high risk of atherosclerotic events, which provides a potential reason for the development of hypertension (76).

### Hypercholesterolemia

Dyslipidemia is an important preventable risk factor for cardiovascular diseases. The accumulation of extra amount of cholesterol in the cells of arterial walls can lead to the formation of vascular atherosclerotic lesions (77). TMAO was found to accelerate the atherosclerotic processes by disturbing lipid metabolism, particularly cholesterol metabolism (78). Physiologically, cellular cholesterol is transferred from the peripheral tissues to the liver and intestine for fecal excretion; however, this pivotal pathway, designated reverse cholesterol transport (RCT), can be inhibited by TMAO. In the animal study, mice fed either choline or TMAO diets showed significant reduction in RCT compared to controlled group, this finding suggested that TMAO inhibited macrophage RCT in vivo (15). Further studies revealed that FMO3, the TMAO‐generating enzyme played a significant role in regulating cholesterol metabolism and RCT. TMA/FMO3/TMAO pathway was identified to be the key integrator of lipid metabolism. FMO3 regulates hepatic liver X receptor (LXR) signaling, which controls inflammation as well as endoplasmic reticulum (ER) stress (79). FMO3 knockdown promotes the nonbiliary macrophage RCT and reorganizes the cholesterol balance, resulting in decreased total plasma cholesterol levels, elevated LDL cholesterol levels and reduced very low‐density lipoprotein (VLDL) cholesterol levels. Interesting, regulating cholesterol balance seen with FMO does not involve TMAO but TMA (79). Another predominant pathway for eliminating the extra cholesterol is hepatic conversion from cholesterol to bile acid, which is ultimately excreted into feces. Disruption of normal bile acid metabolism leads to atherosclerosis. It was showed that TMAO inhibited the hepatic bile acids synthesis by downregulating cholesterol 7α‑hydroxylase (Cyp7a1) expression, which is the key enzyme of this pathway (80). Nevertheless, the exact regulatory mechanism remains to be elucidated, since current concerning about whether the influence of TMAO on the cellular metabolism is direct or indirect remains controversial.

### Diabetic mellitus

Epidemiological studies have suggested that type 2 diabetes mellitus (T2DM) is an independent risk factor for cardiovascular disease, which promotes the progression of atherosclerotic lesions (81). It is characterized by hyperglycemia with relative insulin insufficiency and insulin resistance (IR). The association between T2DM and plasma levels of TMAO was illustrated in multitude of studies. In a large prospective cohort study of healthy participants, the high incident of T2DM was associated with the dietary intakes of phosphatidylcholine, the dietary precursor of TMAO. And the risk of T2D increased by 17% for each 100 mg increase in choline from phosphatidylcholine (82). In a Norwegian epidemiological population study, patients with diabetes presented higher circulating levels of choline than those without. Preclinical studies also showed that diabetic mice had significantly elevated TMAO levels with increased body weight, insulin resistance as compared with nondiabetic mice (83). Mice receiving TMAO dietary gavage exhibited impaired glucose tolerance, indicating that TMAO may inhibit the hepatic insulin signaling pathway and result in adipose tissue inflammation (84).

It was showed that TMAO exacerbated hyperglycemia and IR via phosphatidylinositol 3‐kinase (PI3K)/Akt signaling pathway (85). PI3K plays a key role in delivering insulin signal to regulate glucose metabolism, responsible for the activation of Akt. Akt is the downstream molecule of the PI3K signaling pathway, involved in activation of glycogen synthetase 2 (GYS2), which is a critical enzyme for the storage of hepatic glycogen (84, 85). However, consumption of TMAO reduced mRNA expression of PI3K and Akt, indicating the negative role of TMAO in regulation of glucose homeostasis and development of IR through suppressing insulin signaling pathway (84). Hence, the elimination of TMAO is beneficial to promote the glucose metabolism, which may represent a therapeutic approach for T2DM and atherosclerosis treatments.

### Chronic kidney disease

Atherosclerotic events are more common in patients who had chronic kidney disease (CKD). Accumulating studies showed the high levels of TMAO may account for the greater prevalence of cardiovascular diseases in patients with CKD. In a large cohort of 2529 CKD patients, subjects with higher levels of TMAO were independently associated with increased risk of cardiovascular events (86). Besides, elevated TMAO levels portended the mortality risk in patients with kidney dysfunction. A prospective study of 521 stable CKD patients showed that a high serum TMAO levels were associated with 2.8‐fold increased mortality risk (87). The preclinical data revealed the role of TMAO in impairing renal function. High‐TMAO levels may promote tubulointerstitial fibrosis as well as collagen deposition, and the marker kidney injury molecule‐1 was expressed strikingly (87). In obesity mice, it was found that TMAO exerted a significant effect on renal interstitial fibrosis by aggravating inflammation and oxidative stress (88).

However, there is still controversy on the role of TMAO in kidney dysfunction. As TMAO is a renally excreted small molecular compound, TMAO levels are largely affected by renal function (89). In patients with CKD or end‐stage renal disease, increased TMAO levels are found to be positively correlated with both serum creatine and urea (90). Although TMAO promoting the pathogenesis of CKD was widely accepted, whether the high levels of TMAO in CKD patients are the causes or outcomes of renal dysfunction are still a matter of debates, and more evidence remains to be fully elucidated.

## TREATMENTS TARGETING TMAO

**Fig. 3:**
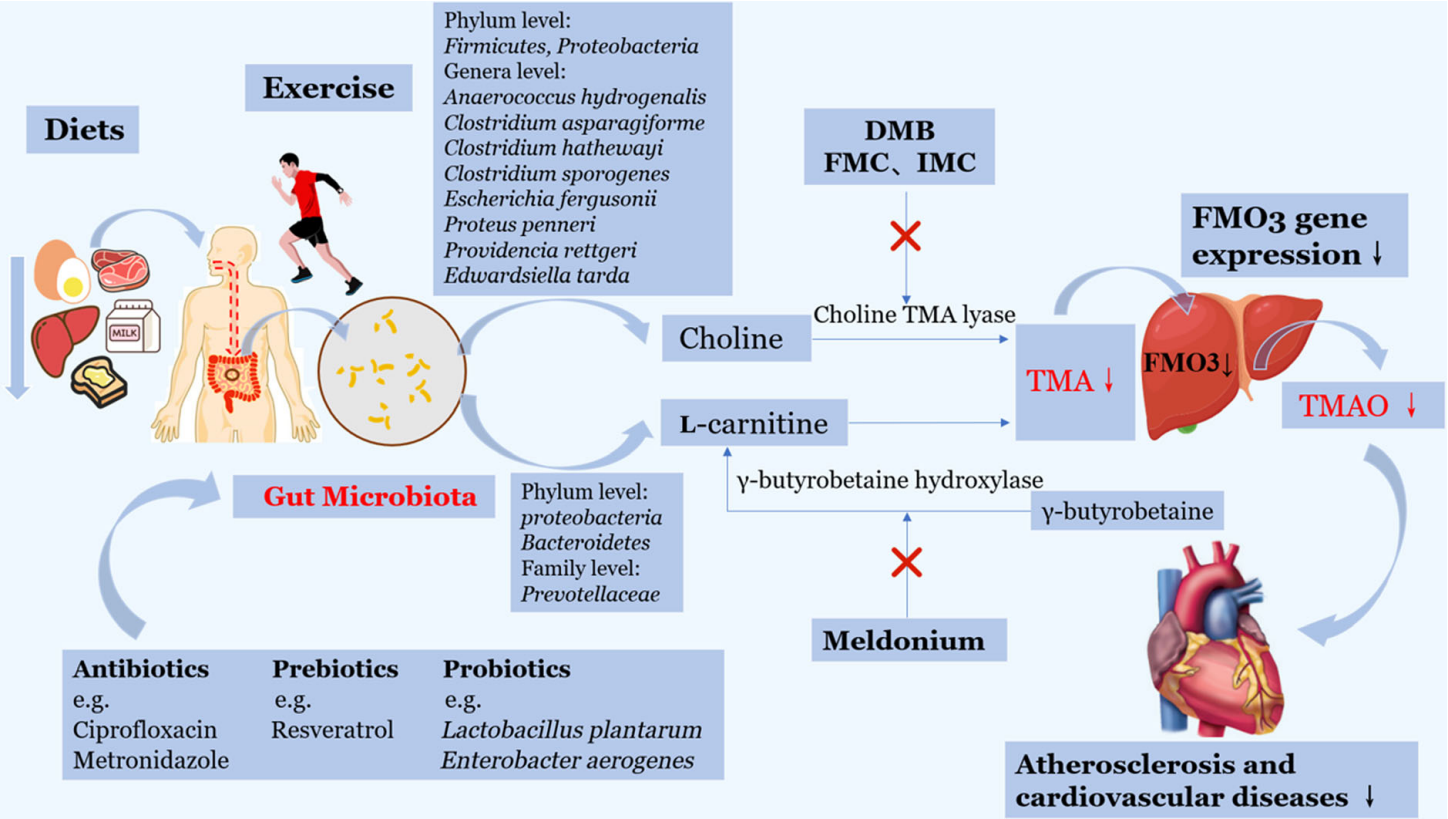
Microbiota‐derived TMAO as a potential therapeutic target for atherosclerotic treatments. Trimethylamine N‐oxide (TMAO) is one of gut microbiota‐derived metabolites, and the approaches that can alter gut microbiota composition may affect TMAO production, including less intake of choline‐rich diets, keeping exercises, administration of certain antibiotics, probiotics and prebiotics. Further, TMA inhibitors including choline TMA lyase inhibitors as well as γ‐butyrobetaine hydroxylase enzyme inhibitors may reduce the levels of TMAO by inhibiting bacterial enzymes. Also, downregulation of flavin monooxygenase 3(FMO3) gene expression can decrease TMAO production. TMAO may become a potential and promising anti‐atherosclerosis target. DMB: 3,3‐dimethyl‐1‐butanol; FMC: Fluoromethylcholine; IMC: iodomethylcholine.

Given that the gut microbiota‐derived TMAO is a detrimental contributor to atherosclerosis and risk factor of CVDs, TMAO is gradually recognized as potential approach for anti‐atherosclerosis therapy. Inhibiting TMAO generation and modifying associated gut microbiota are of great interest. Several advanced therapeutic approaches targeting microbiota‐derived TMAO formation and metabolism have been proposed (Fig. 3).

## INHIBITORS OF ENZYMES IN TMAO FORMATION

Identifying the specific microbial enzymes of TMAO production has triggered the interest of several recent researches. FMO3 is a significant enzyme in the liver that can convert TMA into TMAO, whereas the inhibition of FMO3 leads to accumulation of TMA, which induces trimethylaminuria, also known as fish malodor syndrome (91, 92). Thus, the optimal approach for reduction of TMAO production is to target the production of TMA directly. In particular, the significant portion of TMAO production is likely accounted for microbial conversion of choline into TMA. Inhibiting this conversion via organismal pathway is a potential therapy for the decrease of TMAO generation (93).

3,3‐dimethyl‐1‐butanol (DMB), the structural analog of choline, which serves as a tool drug to lower TMA formation through inhibition of microbial choline TMA lyse (94). It was showed to significantly reduce the circulating levels of TMAO non‐lethally in ApoE−/− mice fed a high choline diet (94). Importantly, DMB suppressed the high choline‐diet induced endogenous macrophage foam cell formation and ameliorated atherosclerotic lesions development in vivo (94). In line with previous studies, DMB was detected to attenuate platelet activation and reduce the rate of thrombus formation (93). Despite these promising results, these processes were reversible, which meant that direct injection of TMAO could completely reverse DMB‐dependent inhibition in rate of thrombus formation (93). Fluoromethylcholine (FMC) and iodomethylcholine (IMC) are second‐generation TMA lyase inhibitors and choline analogues. In contrast to DMB, both FMC and IMC promoted the irreversible inhibition of microbial TMA lyase via generating a reactive species, which had the greatly tight interaction with active site residue of TMA lyase after C‐N bond cleavage (93). Remarkably, they displayed enhanced inhibitory potency, including suppressing TMA and TMAO levels and reducing the rate of thrombus formation without observed toxicity in vivo compared with DMB (93).

Another example is the pharmacological agent Meldonium, which is the analogue of γ‐butyrobetaine. Emerging data showed that chronic administration of Meldonium decreased the circulating levels of L‐carnitine in healthy nonvegetarian individuals via suppressing γ‐butyrobetaine hydroxylase enzyme (95, 96). Study also found that treatment with Meldonium reduced the plasma concentration of TMAO by increasing urinary excretion (97, 98). These inhibitors of the TMAO production may shed new light on the treatment or even prevention of the atherosclerosis associated with cardiovascular diseases.

### Diet and exercise

Several lifestyle factors such as diet and exercise may exert profound effects on the shift in gut microbial composition and evidently affect gut microbiota‐derived TMAO production. Regarding different dietary preferences, previous studies have indicated that individuals on omnivorous diets have remarkably synthetic capacity to generate TMAO from the diet, exhibiting higher plasma TMAO levels than the vegetarians following ingestion of L‐carnitine (15). Supplementation of L‐carnitine in animal model also showed that elevated production of TMA and TMAO by 10‐fold with altered gut microbiota composition (99, 100). Unlike the L‐carnitine, choline is an essential nutrient and cannot be eliminated thoroughly from the body. It is the precursor of neurotransmitter acetylcholine and mainly forms the cellular membrane, participating in cholesterol transportation (101). Recent studies have suggested that ingestion of choline‐rich diet yielded significantly high levels of TMAO (20, 102). Thus, it might be preferable to consume less choline to reduce TMAO.

In addition to diets, exercise has been shown to change gut microbial composition and diversity (103, 104). Recent research has indicated that supervised exercise combined with caloric restricted diets effectively lowered the TMAO levels (105). Given the role of TMAO in atherosclerosis, exercise is a significant leading approach that may slow the progression of atherosclerosis and reduce risks of cardiovascular diseases.

### Genetic determinants

Genetic factors may play an intrinsic role in determining TMAO levels. The gene of FMO3 is by far the most active and effective FMO enzyme family member that can convert TMA to TMAO (106). It is also the only gene found to alter the circulating levels of TMAO in humans. FMO3 is located on chromosome 1q24.3 and consists of 10 exons responsible for encoding a 532‐residue enzyme (107). The mutation deficiency of FMO3 results in a rare disease named trimethylaminuria, which is characterized by the failure to break down TMA. FMO3 deficiency and antisense‐mediated silence of FMO3 can reduce TMAO levels, while overexpression of hepatic FMO3 can enhance TMAO generation (17). FMO3 knockout mice showed decreased TMAO levels, coinciding with reduced platelet activation and thrombogenic potential (108). Besides, FMO3 expression is affected by sex hormones via regulation of farnesoid X receptor (FXR) in vivo. Specifically, androgens can downregulate FMO3 expression with the finding that female mice had greater susceptibility to develop atherosclerosis than male mice (17). However, in humans, the expression of FMO3 and circulating TMAO levels did not show sexual dimorphism (17, 20). The discordant results and variation in TMAO may be due to the more important determinants, such as various dietary preference and gut microbiota metabolism (107).

### Antibiotics

Given that the biosynthesis and production of TMAO relying on the gut microbial metabolism, antibiotics are regarded as a promising approach to target microbiota‐derived TMAO production. In a clinical study, healthy participants were required to administrate ciprofloxacin plus metronidazole for one month, which are broad‐spectrum antibiotics. It was found that administration of antibiotics could effectively decrease plasma levels of TMAO. However, after the withdrawal of antibiotics, the plasma TMAO levels returned to the initial levels with recovery of intestinal microbiota (20). Long‐term antibiotic exposure can lead to the resistance of intestinal microbial colonization in the hosts, and the TMAO levels gradually rebounded to the initial levels (13). More notably, antibiotics can not only target harmful bacteria, but also some beneficial species. The use of antibiotics for long term can induce gut microbial dysbiosis and affect the health of hosts, which limit the use of antibiotics as a practical therapy for anti‐atherosclerosis. Further, no meta‐analysis of clinical randomized controlled trials confirmed the efficacy of antibiotics in decreasing the mortality of cardiovascular events (109).

### Prebiotics and probiotics

Prebiotics are nondigestible dietary ingredients that can be fermented by specific intestinal microflora to stimulate the growth of beneficial bacteria colonized in the gut. Probiotics are live and natural microbiota that can be administrated safely (110, 111). Both prebiotics and probiotics are good candidates for the modulating the gut microbiota and conferring favorable effects to the host. An example of prebiotics is as follows: Resveratrol is a natural phenolic phytochemical with poor bioavailability in the intestinal, and it can modulate intestinal bacteria effectively. Intake of Resveratrol promotes the growth of beneficial bacteria including *Bacteroides, Lactobacillus and Bifidobacterium* with the decline in the TMAO levels (112, 113). Preclinical data have showed that Resveratrol decreased TMAO levels and inhibited development of atherosclerosis in vivo (114). As with prebiotics, the consumption of various probiotics such as *Lactobacillus plantarum* could decrease TMAO production and attenuate atherosclerotic lesion formation in ApoE^−/−^ mice (111). Another example of probiotics is *E. aerogenes*, the serum TMAO and fecal TMA levels in mice fed choline‐rich diet with *E. aerogenes* were lower compared with those of control group. The study showed that treatment with *E. aerogenes* significantly decreased serum TMAO levels with altered microbial composition, which might be an alternative approach for atherosclerosis treatment (115). Besides that, there is a group of methanogens colonized in the gastrointestinal tract that can only utilize TMA as a substrate. A recent research has found that the *Methanomassiliicoccus luminyensis strain B10* was able to deplete TMA and TMAO by combining H_2_ for methanogenesis (116). Although convincing evidence emerged from preclinical studies, these strategies focusing on gut microbiota and microbial metabolites provide promising insight into treatment of atherosclerosis and cardiovascular diseases.

## PERSPECTIVE AND CONCLUSION

Recently, gut microbiota and microbial metabolites, particularly TMAO, have gained much attention for the potential role in promoting atherosclerosis. Innovative therapeutic approaches targeting gut microbiota and TMAO, including lifestyle modifications, TMA and FMO3 inhibitors, antibiotics, prebiotics and probiotics, have shed novel light on treating atherosclerosis and cardiovascular diseases. Currently, the underlying associations between TMAO and development of atherosclerosis have been illuminated in numerous previous works. Although the findings were promising, the exact mechanism of gut dysbiosis and microbiota‐derived TMAO involved in atherosclerosis may still be the tip of the iceberg. More well‐conducted studies focusing on molecular mechanisms and precise treatments targeting gut microbiota‐dependent metabolites for anti‐atherosclerosis remain to be further explored and fully clarified.

We are thankful to Important Weak Subject Construction Project of Pudong Health and Family Planning Commission of Shanghai (No. PWZbr2017‐06) and Scientific Research Project of Shanghai Municipal Health and Family Planning Commission (No. 20184Y0213) for providing financial assistance.

## CONFLICT OF INTEREST

The authors declare no conflict of interest.

## References

[1] Benjamin EJ , Blaha MJ , Chiuve SE , Cushman M , Das SR , Deo R , et al. Heart disease and stroke statistics‐2017 update: a report from the American heart association. Circulation 2017;135:e146–603. 28122885 10.1161/CIR.0000000000000485 PMC5408160

[2] da Silva RM . Influence of inflammation and atherosclerosis in atrial fibrillation. Curr Atheroscler Rep 2017;19:2. 28102478 10.1007/s11883-017-0639-0

[3] Campbell LA , Rosenfeld ME . Infection and atherosclerosis development. Arch Med Res 2015;46:339–50. 26004263 10.1016/j.arcmed.2015.05.006 PMC4524506

[4] Cui Y , Lv X , Wang F , Kong J , Zhao H , Ye Z , et al. Geometry of the carotid artery and its association with pathologic changes in a Chinese population. Front Physiol 2019;10:1628. 32038300 10.3389/fphys.2019.01628 PMC6985580

[5] Fihn SD , Gardin JM , Abrams J , Berra K , Blankenship JC , Dallas AP , et al. 2012 ACCF/AHA/ACP/AATS/PCNA/SCAI/STS Guideline for the diagnosis and management of patients with stable ischemic heart disease: a report of the American College of Cardiology Foundation/American Heart Association Task Force on Practice Guidelines, and the American College of Physicians, American Association for Thoracic Surgery, Preventive Cardiovascular Nurses Association, Society for Cardiovascular Angiography and Interventions, and Society of Thoracic Surgeons. J Am Coll Cardiol 2012;60:e44–164. 23182125 10.1016/j.jacc.2012.07.013

[6] Fruchart JC , Sacks F , Hermans MP , Assmann G , Brown WV , Ceska R , et al. The residual risk reduction initiative: a call to action to reduce residual vascular risk in patients with dyslipidemia. Am J Cardiol 2008;102:1k–34k. 19068318 10.1016/S0002-9149(08)01833-X

[7] Wang JZ , Du WT , Xu YL , Cheng SZ , Liu ZJ . Gut microbiome‐based medical methodologies for early‐stage disease prevention. Microb Pathog 2017;105:122–30. 28219830 10.1016/j.micpath.2017.02.024

[8] Stilling RM , Dinan TG , Cryan JF . Microbial genes, brain & behaviour ‐ epigenetic regulation of the gut‐brain axis. Genes Brain Behav 2014;13:69–86. 24286462 10.1111/gbb.12109

[9] Wang Z , Klipfell E , Bennett BJ , Koeth R , Levison BS , Dugar B , et al. Gut flora metabolism of phosphatidylcholine promotes cardiovascular disease. Nature 2011;472:57–63. 21475195 10.1038/nature09922 PMC3086762

[10] Ahmadmehrabi S , Tang WHW . Gut microbiome and its role in cardiovascular diseases. Curr Opin Cardiol 2017;32(6):761–6. 29023288 10.1097/HCO.0000000000000445 PMC5746314

[11] Yamashita T . Intestinal immunity and gut microbiota in atherogenesis. J Atheroscler Thromb 2017;24:110–9. 27928097 10.5551/jat.38265 PMC5305671

[12] Ascher S , Reinhardt C . The gut microbiota: An emerging risk factor for cardiovascular and cerebrovascular disease. Eur J Immunol 2018;48:564–75. 29230812 10.1002/eji.201646879

[13] Jie Z , Xia H , Zhong SL , Feng Q , Li S , Liang S , et al. The gut microbiome in atherosclerotic cardiovascular disease. Nat Commun 2017;8:845. 29018189 10.1038/s41467-017-00900-1 PMC5635030

[14] Ufnal M , Zadlo A , Ostaszewski R . TMAO: a small molecule of great expectations. Nutrition 2015;31:1317–23. 26283574 10.1016/j.nut.2015.05.006

[15] Koeth RA , Wang Z , Levison BS , Buffa JA , Org E , Sheehy BT , et al. Intestinal microbiota metabolism of L‐carnitine, a nutrient in red meat, promotes atherosclerosis. Nat Med 2013;19:576–85. 23563705 10.1038/nm.3145 PMC3650111

[16] Ussher JR , Lopaschuk GD , Arduini A . Gut microbiota metabolism of L‐carnitine and cardiovascular risk. Atherosclerosis 2013;231:456–61. 24267266 10.1016/j.atherosclerosis.2013.10.013

[17] Bennett BJ , de Aguiar Vallim TQ , Wang Z , Shih DM , Meng Y , Gregory J , et al. Trimethylamine‐N‐oxide, a metabolite associated with atherosclerosis, exhibits complex genetic and dietary regulation. Cell Metab 2013;17:49–60. 23312283 10.1016/j.cmet.2012.12.011 PMC3771112

[18] Gregory JC , Buffa JA , Org E , Wang Z , Levison BS , Zhu W , et al. Transmission of atherosclerosis susceptibility with gut microbial transplantation. J Biol Chem 2015;290:5647–60. 25550161 10.1074/jbc.M114.618249 PMC4342477

[19] Wang Z , Tang WH , Buffa JA , Fu X , Britt EB , Koeth RA , et al. Prognostic value of choline and betaine depends on intestinal microbiota‐generated metabolite trimethylamine‐N‐oxide. Eur Heart J 2014;35:904–10. 24497336 10.1093/eurheartj/ehu002 PMC3977137

[20] Tang WH , Wang Z , Levison BS , Koeth RA , Britt EB , Fu X , et al. Intestinal microbial metabolism of phosphatidylcholine and cardiovascular risk. N Engl J Med 2013;368:1575–84. 23614584 10.1056/NEJMoa1109400 PMC3701945

[21] Nie J , Xie L , Zhao BX , Li Y , Qiu B , Zhu F , et al. Serum trimethylamine N‐oxide concentration is positively associated with first stroke in hypertensive patients. Stroke 2018;49:2021–8. 30354996 10.1161/STROKEAHA.118.021997

[22] Randrianarisoa E , Lehn‐Stefan A , Wang X , Hoene M , Peter A , Heinzmann SS , et al. Relationship of serum trimethylamine N‐oxide (TMAO) levels with early atherosclerosis in humans. Sci Rep 2016;6:26745. 27228955 10.1038/srep26745 PMC4882652

[23] Senthong V , Wang Z , Fan Y , Wu Y , Hazen SL , Tang WH . Trimethylamine N‐oxide and mortality risk in patients with peripheral artery disease. J Am Heart Assoc 2016;5:e004237. 10.1161/JAHA.116.004237 PMC5121520 27792653

[24] Li XS , Obeid S , Klingenberg R , Gencer B , Mach F , Raber L , et al. Gut microbiota‐dependent trimethylamine N‐oxide in acute coronary syndromes: a prognostic marker for incident cardiovascular events beyond traditional risk factors. Eur Heart J 2017;38:814–24. 28077467 10.1093/eurheartj/ehw582 PMC5837488

[25] Zeisel SH , Warrier M . Trimethylamine N‐oxide, the microbiome, and heart and kidney disease. Annu Rev Nutr 2017;37:157–81. 28715991 10.1146/annurev-nutr-071816-064732

[26] Tomlinson JAP , Wheeler DC . The role of trimethylamine N‐oxide as a mediator of cardiovascular complications in chronic kidney disease. Kidney Int 2017;92:809–15. 28807612 10.1016/j.kint.2017.03.053

[27] Chhibber‐Goel J , Gaur A , Singhal V , Parakh N , Bhargava B , Sharma A . The complex metabolism of trimethylamine in humans: endogenous and exogenous sources. Expert Rev Mol Med 2016;18:e8. 27126549 10.1017/erm.2016.6

[28] Wallace TC , Blusztajn JK , Caudill MA , Klatt KC , Natker E , Zeisel SH , et al. Choline: the underconsumed and underappreciated essential nutrient. Nutr Today 2018;53:240–53. 30853718 10.1097/NT.0000000000000302 PMC6259877

[29] Zeisel SH , Mar MH , Howe JC , Holden JM . Concentrations of choline‐containing compounds and betaine in common foods. J Nutr 2003;133:1302–7. 12730414 10.1093/jn/133.5.1302

[30] Zeisel SH , da Costa KA . Choline: an essential nutrient for public health. Nutr Rev 2009;67:615–23. 19906248 10.1111/j.1753-4887.2009.00246.x PMC2782876

[31] Fennema D , Phillips IR , Shephard EA . Trimethylamine and trimethylamine N‐Oxide, a flavin‐containing monooxygenase 3 (FMO3)‐mediated host‐microbiome metabolic axis implicated in health and disease. Drug Metab Dispos 2016;44:1839–50. 27190056 10.1124/dmd.116.070615 PMC5074467

[32] Walker JA , Friesen JD , Peters SJ , Jones MA , Friesen JA . Development of a new and reliable assay for choline kinase using (31)P NMR. Heliyon 2019;5:e02585. 31687487 10.1016/j.heliyon.2019.e02585 PMC6820101

[33] Zhu Y , Jameson E , Crosatti M , Schafer H , Rajakumar K , Bugg TD , et al. Carnitine metabolism to trimethylamine by an unusual Rieske‐type oxygenase from human microbiota. Proc Natl Acad Sci USA 2014;111:4268–73. 24591617 10.1073/pnas.1316569111 PMC3964110

[34] Rebouche CJ , Seim H . Carnitine metabolism and its regulation in microorganisms and mammals. Annu Rev Nutr 1998;18:39–61. 9706218 10.1146/annurev.nutr.18.1.39

[35] Koeth RA , Levison BS , Culley MK , Buffa JA , Wang Z , Gregory JC , et al. Gamma‐Butyrobetaine is a proatherogenic intermediate in gut microbial metabolism of L‐carnitine to TMAO. Cell Metab 2014;20:799–812. 25440057 10.1016/j.cmet.2014.10.006 PMC4255476

[36] Zhang AQ , Mitchell SC , Smith RL . Dietary precursors of trimethylamine in man: a pilot study. Food Chem Toxicol 1999;37:515–20. 10456680 10.1016/s0278-6915(99)00028-9

[37] Romano KA , Vivas EI , Amador‐Noguez D , Rey FE . Intestinal microbiota composition modulates choline bioavailability from diet and accumulation of the proatherogenic metabolite trimethylamine‐N‐oxide. mBio 2015;6:e02481. 25784704 10.1128/mBio.02481-14 PMC4453578

[38] Al‐Waiz M , Mikov M , Mitchell Sc , Smith Rl . The exogenous origin of trimethylamine in the mouse. Metabolism 1992;41:135–6. 1736035 10.1016/0026-0495(92)90140-6

[39] Oellgaard J , Winther SA , Hansen TS , Rossing P , von Scholten BJ . Trimethylamine N‐oxide (TMAO) as a new potential therapeutic target for insulin resistance and cancer. Curr Pharm Des 2017;23:3699–712. 28641532 10.2174/1381612823666170622095324

[40] Senthong V , Wang Z , Li XS , Fan Y , Wu Y , Tang WH , et al. Intestinal microbiota‐generated metabolite trimethylamine‐N‐oxide and 5‐year mortality risk in stable coronary artery disease: the contributory role of intestinal microbiota in a COURAGE‐Like patient cohort. J Am Heart Assoc 2016;5:e002816. 10.1161/JAHA.115.002816 PMC4937244 27287696

[41] Troseid M , Ueland T , Hov JR , Svardal A , Gregersen I , Dahl CP , et al. Microbiota‐dependent metabolite trimethylamine‐N‐oxide is associated with disease severity and survival of patients with chronic heart failure. J Intern Med 2015;277:717–26. 25382824 10.1111/joim.12328

[42] Tang WH , Wang Z , Shrestha K , Borowski AG , Wu Y , Troughton RW , et al. Intestinal microbiota‐dependent phosphatidylcholine metabolites, diastolic dysfunction, and adverse clinical outcomes in chronic systolic heart failure. J Card Fail 2015;21:91–6. 25459686 10.1016/j.cardfail.2014.11.006 PMC4312712

[43] Stubbs JR , House JA , Ocque AJ , Zhang S , Johnson C , Kimber C , et al. Serum trimethylamine‐N‐oxide is elevated in CKD and correlates with coronary atherosclerosis burden. J Am Soc Nephrol 2016;27:305–13. 26229137 10.1681/ASN.2014111063 PMC4696571

[44] Haghikia A , Li XS , Liman TG , Bledau N , Schmidt D , Zimmermann F , et al. Gut microbiota‐dependent trimethylamine N‐oxide predicts risk of cardiovascular events in patients with stroke and is related to proinflammatory monocytes. Arterioscler Thromb Vasc Biol 2018;38:2225–35. 29976769 10.1161/ATVBAHA.118.311023 PMC6202215

[45] Suzuki T , Heaney LM , Jones DJ , Ng LL . Trimethylamine N‐oxide and risk stratification after acute myocardial infarction. Clin Chem 2017;63:420–8. 28062632 10.1373/clinchem.2016.264853

[46] Lever M , George PM , Slow S , Bellamy D , Young JM , Ho M , et al. Betaine and trimethylamine‐N‐oxide as predictors of cardiovascular outcomes show different patterns in diabetes mellitus: an observational study. PLoS One 2014;9:e114969. 25493436 10.1371/journal.pone.0114969 PMC4262445

[47] Schiattarella GG , Sannino A , Toscano E , Giugliano G , Gargiulo G , Franzone A , et al. Gut microbe‐generated metabolite trimethylamine‐N‐oxide as cardiovascular risk biomarker: a systematic review and dose‐response meta‐analysis. Eur Heart J 2017;38:2948–56. 29020409 10.1093/eurheartj/ehx342

[48] Yamaura Y , Watanabe N , Obase K , Hayashida A , Okura H , Yoshida K . Relation between progression of aortic valve sclerosis and carotid intima‐media thickening in asymptomatic subjects with cardiovascular risk factors. J Echocardiogr 2010;8:87–93. 27278799 10.1007/s12574-010-0038-9

[49] Lorenz MW , Markus HS , Bots ML , Rosvall M , Sitzer M . Prediction of clinical cardiovascular events with carotid intima‐media thickness: a systematic review and meta‐analysis. Circulation 2007;115:459–67. 17242284 10.1161/CIRCULATIONAHA.106.628875

[50] Hodis HN , Mack WJ , LaBree L , Selzer RH , Liu CR , Liu CH , et al. The role of carotid arterial intima‐media thickness in predicting clinical coronary events. Ann Intern Med 1998;128:262–9. 9471928 10.7326/0003-4819-128-4-199802150-00002

[51] Skagen K , Troseid M , Ueland T , Holm S , Abbas A , Gregersen I , et al. The carnitine‐butyrobetaine‐trimethylamine‐N‐oxide pathway and its association with cardiovascular mortality in patients with carotid atherosclerosis. Atherosclerosis 2016;247:64–9. 26868510 10.1016/j.atherosclerosis.2016.01.033

[52] Meyer KA , Benton TZ , Bennett BJ , Jacobs DR Jr , Lloyd‐Jones DM , Gross MD , et al. Microbiota‐dependent metabolite trimethylamine N‐oxide and coronary artery calcium in the coronary artery risk development in young adults study (CARDIA). J Am Heart Assoc 2016;5:e003970. 10.1161/JAHA.116.003970 PMC5121500 27792658

[53] Chistiakov DA , Melnichenko AA , Myasoedova VA , Grechko AV , Orekhov AN . Mechanisms of foam cell formation in atherosclerosis. J Mol Med 2017;95:1153–65. 28785870 10.1007/s00109-017-1575-8

[54] Thon MP , Hemmler A , Glinzer A , Mayr M , Wildgruber M , Zernecke‐Madsen A , et al. A multiphysics approach for modeling early atherosclerosis. Biomech Model Mechanobiol 2018;17:617–44. 29159532 10.1007/s10237-017-0982-7

[55] Geng J , Yang C , Wang B , Zhang X , Hu T , Gu Y , et al. Trimethylamine N‐oxide promotes atherosclerosis via CD36‐dependent MAPK/JNK pathway. Biomed Pharmacother 2018;97:941–7. 29136772 10.1016/j.biopha.2017.11.016

[56] Mohammadi A , Najar AG , Yaghoobi MM , Jahani Y , Vahabzadeh Z . Trimethylamine‐N‐oxide treatment induces changes in the ATP‐binding cassette transporter A1 and scavenger receptor A1 in murine macrophage J774A.1 cells. Inflammation 2016;39:393–404. 26412259 10.1007/s10753-015-0261-7

[57] Sun X , Jiao X , Ma Y , Liu Y , Zhang L , He Y , et al. Trimethylamine N‐oxide induces inflammation and endothelial dysfunction in human umbilical vein endothelial cells via activating ROS‐TXNIP‐NLRP3 inflammasome. Biochem Biophys Res Commun 2016;481:63–70. 27833015 10.1016/j.bbrc.2016.11.017

[58] Ren D , Liu Y , Zhao Y , Yang X . Hepatotoxicity and endothelial dysfunction induced by high choline diet and the protective effects of phloretin in mice. Food Chem Toxicol 2016;94:203–12. 27316781 10.1016/j.fct.2016.06.004

[59] Chou RH , Chen CY , Chen IC , Huang HL , Lu YW , Kuo CS , et al. Trimethylamine N‐oxide, circulating endothelial progenitor cells, and endothelial function in patients with stable angina. Sci Rep 2019;9:4249. 30862856 10.1038/s41598-019-40638-y PMC6414518

[60] Seldin MM , Meng Y , Qi H , Zhu W , Wang Z , Hazen SL , et al. Trimethylamine N‐oxide promotes vascular inflammation through signaling of mitogen‐activated protein kinase and nuclear factor‐kappaB. J Am Heart Assoc 2016;5:e002767. 26903003 10.1161/JAHA.115.002767 PMC4802459

[61] Chen H , Li J , Li N , Liu H , Tang J . Increased circulating trimethylamine N‐oxide plays a contributory role in the development of endothelial dysfunction and hypertension in the RUPP rat model of preeclampsia. Hypertens Pregnancy 2019;38:96–104. 30821524 10.1080/10641955.2019.1584630

[62] George TW , Waroonphan S , Niwat C , Gordon MH , Lovegrove JA . The Glu298Asp single nucleotide polymorphism in the endothelial nitric oxide synthase gene differentially affects the vascular response to acute consumption of fruit and vegetable puree based drinks. Mol Nutr Food Res 2012;56:1014–24. 22689471 10.1002/mnfr.201100689

[63] Ma G , Pan B , Chen Y , Guo C , Zhao M , Zheng L , et al. Trimethylamine N‐oxide in atherogenesis: impairing endothelial self‐repair capacity and enhancing monocyte adhesion. Biosci Rep 2017;37:BSR20160244 28153917 10.1042/BSR20160244 PMC5333780

[64] Rai V , Agrawal DK . The role of damage‐ and pathogen‐associated molecular patterns in inflammation‐mediated vulnerability of atherosclerotic plaques. Can J Physiol Pharmacol 2017;95:1245–53. 28746820 10.1139/cjpp-2016-0664

[65] Fuster V , Badimon L , Badimon JJ , Chesebro JH . The pathogenesis of coronary artery disease and the acute coronary syndromes. N Engl J Med 1992;326:310–8. 1728735 10.1056/NEJM199201303260506

[66] Falk E , Shah PK , Fuster V . Coronary plaque disruption. Circulation 1995;92:657–71. 7634481 10.1161/01.cir.92.3.657

[67] Fu Q , Zhao M , Wang D , Hu H , Guo C , Chen W , et al. Coronary plaque characterization assessed by optical coherence tomography and plasma trimethylamine‐N‐oxide levels in patients with coronary artery disease. Am J Cardiol 2016;118:1311–5. 27600460 10.1016/j.amjcard.2016.07.071

[68] Liu X , Xie Z , Sun M , Wang X , Li J , Cui J , et al. Plasma trimethylamine N‐oxide is associated with vulnerable plaque characteristics in CAD patients as assessed by optical coherence tomography. Int J Cardiol 2018;265:18–23. 29729869 10.1016/j.ijcard.2018.04.126

[69] Virmani R , Kolodgie FD , Burke AP , Finn AV , Gold HK , Tulenko TN , et al. Atherosclerotic plaque progression and vulnerability to rupture: angiogenesis as a source of intraplaque hemorrhage. Arterioscler Thromb Vasc Biol 2005;25:2054–61. 16037567 10.1161/01.ATV.0000178991.71605.18

[70] Zhu W , Gregory JC , Org E , Buffa JA , Gupta N , Wang Z , et al. Gut microbial metabolite TMAO enhances platelet hyperreactivity and thrombosis risk. Cell 2016;165:111–24. 26972052 10.1016/j.cell.2016.02.011 PMC4862743

[71] Zhu W , Wang Z , Tang WHW , Hazen SL . Gut microbe‐generated trimethylamine N‐oxide from dietary choline is prothrombotic in subjects. Circulation 2017;135:1671–3. 28438808 10.1161/CIRCULATIONAHA.116.025338 PMC5460631

[72] Furie B , Furie BC . Mechanisms of thrombus formation. N Engl J Med 2008;359:938–49. 18753650 10.1056/NEJMra0801082

[73] Yang T , Santisteban MM , Rodriguez V , Li E , Ahmari N , Carvajal JM , et al. Gut dysbiosis is linked to hypertension. Hypertension 2015;65:1331–40. 25870193 10.1161/HYPERTENSIONAHA.115.05315 PMC4433416

[74] Kang Y , Cai Y . Gut microbiota and hypertension: From pathogenesis to new therapeutic strategies. Clin Res Hepatol Gastroenterol 2018;42:110–7. 29102544 10.1016/j.clinre.2017.09.006

[75] Ufnal M , Jazwiec R , Dadlez M , Drapala A , Sikora M , Skrzypecki J . Trimethylamine‐N‐oxide: a carnitine‐derived metabolite that prolongs the hypertensive effect of angiotensin II in rats. Can J Cardiol 2014;30:1700–5. 25475471 10.1016/j.cjca.2014.09.010

[76] Jaworska K , Huc T , Samborowska E , Dobrowolski L , Bielinska K , Gawlak M , et al. Hypertension in rats is associated with an increased permeability of the colon to TMA, a gut bacteria metabolite. PLoS One 2017;12:e0189310. 29236735 10.1371/journal.pone.0189310 PMC5728578

[77] Chiang JYL , Vlahcevic ZR . The regulation of cholesterol conversion to bile acids. Physiological functions of cytochrome P450 in relation to structure and regulation. Adv Mol Cell Biol 1996;14:269–316.

[78] Trenteseaux C , Gaston AT , Aguesse A , Poupeau G , de Coppet P , Andriantsitohaina R , et al. Perinatal hypercholesterolemia exacerbates atherosclerosis lesions in offspring by altering metabolism of trimethylamine‐N‐oxide and bile acids. Arterioscler Thromb Vasc Biol 2017;37:2053–63. 28935756 10.1161/ATVBAHA.117.309923

[79] Warrier M , Shih DM , Burrows AC , Ferguson D , Gromovsky AD , Brown AL , et al. The TMAO‐generating enzyme flavin monooxygenase 3 is a central regulator of cholesterol balance. Cell Rep 2015;10:326–8. 25600868 10.1016/j.celrep.2014.12.036 PMC4501903

[80] Ding L , Chang M , Guo Y , Zhang L , Xue C , Yanagita T , et al. Trimethylamine‐N‐oxide (TMAO)‐induced atherosclerosis is associated with bile acid metabolism. Lipids Health Dis 2018;17:286. 30567573 10.1186/s12944-018-0939-6 PMC6300890

[81] Ross S , Gerstein H , Pare G . The genetic link between diabetes and atherosclerosis. Can J Cardiol 2018;34:565–74. 29731020 10.1016/j.cjca.2018.01.016

[82] Li Y , Wang DD , Chiuve SE , Manson JE , Willett WC , Hu FB , et al. Dietary phosphatidylcholine intake and type 2 diabetes in men and women. Diabetes Care 2015;38:e13–4. 25614692 10.2337/dc14-2093 PMC4302257

[83] Dambrova M , Latkovskis G , Kuka J , Strele I , Konrade I , Grinberga S , et al. Diabetes is Associated with Higher Trimethylamine N‐oxide Plasma Levels. Exp Clin Endocrinol Diabetes 2016;124:251–6. 27123785 10.1055/s-0035-1569330

[84] Gao X , Liu X , Xu J , Xue C , Xue Y , Wang Y . Dietary trimethylamine N‐oxide exacerbates impaired glucose tolerance in mice fed a high fat diet. J Biosci Bioeng 2014;118:476–81. 24721123 10.1016/j.jbiosc.2014.03.001

[85] Han JW , Zhan XR , Li XY , Xia B , Wang YY , Zhang J , et al. Impaired PI3K/Akt signal pathway and hepatocellular injury in high‐fat fed rats. World J Gastroenterol 2010;16:6111–8. 21182226 10.3748/wjg.v16.i48.6111 PMC3012583

[86] Kim RB , Morse BL , Djurdjev O , Tang M , Muirhead N , Barrett B , et al. Advanced chronic kidney disease populations have elevated trimethylamine N‐oxide levels associated with increased cardiovascular events. Kidney Int 2016;89:1144–52. 27083288 10.1016/j.kint.2016.01.014

[87] Tang WH , Wang Z , Kennedy DJ , Wu Y , Buffa JA , Agatisa‐Boyle B , et al. Gut microbiota‐dependent trimethylamine N‐oxide (TMAO) pathway contributes to both development of renal insufficiency and mortality risk in chronic kidney disease. Circ Res 2015;116:448–55. 25599331 10.1161/CIRCRESAHA.116.305360 PMC4312512

[88] Sun G , Yin Z , Liu N , Bian X , Yu R , Su X , et al. Gut microbial metabolite TMAO contributes to renal dysfunction in a mouse model of diet‐induced obesity. Biochem Biophys Res Commun 2017;493:964–70. 28942145 10.1016/j.bbrc.2017.09.108

[89] Al‐Waiz M , Mitchell SC , Idle JR , Smith RL . The metabolism of 14C‐labelled trimethylamine and its N‐oxide in man. Xenobiotica 1987;17:551–8. 3604260 10.3109/00498258709043962

[90] Bell JD , Lee JA , Lee HA , Sadler PJ , Wilkie DR , Woodham RH . Nuclear magnetic resonance studies of blood plasma and urine from subjects with chronic renal failure: identification of trimethylamine‐N‐oxide. Biochim Biophys Acta 1991;1096:101–7. 2001424 10.1016/0925-4439(91)90046-c

[91] Messenger J , Clark S , Massick S , Bechtel M . A review of trimethylaminuria: (fish odor syndrome). J Clin Aesthet Dermatol 2013;6:45–48. PMC3848652 24307925

[92] Cashman JR , Camp K , Fakharzadeh SS , Fennessey PV , Hines RN , Mamer OA , et al. Biochemical and clinical aspects of the human flavin‐containing monooxygenase form 3 (FMO3) related to trimethylaminuria. Curr Drug Metab 2003;4:151–70. 12678693 10.2174/1389200033489505

[93] Roberts AB , Gu X , Buffa JA , Hurd AG , Wang Z , Zhu W , et al. Development of a gut microbe‐targeted nonlethal therapeutic to inhibit thrombosis potential. Nat Med 2018;24:1407–17. 30082863 10.1038/s41591-018-0128-1 PMC6129214

[94] Wang Z , Roberts AB , Buffa JA , Levison BS , Zhu W , Org E , et al. Non‐lethal inhibition of gut microbial trimethylamine production for the treatment of atherosclerosis. Cell 2015;163:1585–95. 26687352 10.1016/j.cell.2015.11.055 PMC4871610

[95] Liepinsh E , Konrade I , Skapare E , Pugovics O , Grinberga S , Kuka J , et al. Mildronate treatment alters gamma‐butyrobetaine and l‐carnitine concentrations in healthy volunteers. J Pharm Pharmacol 2011;63:1195–201. 21827492 10.1111/j.2042-7158.2011.01325.x

[96] Simkhovich BZ , Shutenko ZV , Meirena DV , Khagi KB , Mezapuke RJ , Molodchina TN , et al. 3‐(2,2,2‐Trimethylhydrazinium)propionate (THP)–a novel gamma‐butyrobetaine hydroxylase inhibitor with cardioprotective properties. Biochem Pharmacol 1988;37:195–202. 3342076 10.1016/0006-2952(88)90717-4

[97] Kuka J , Liepinsh E , Makrecka‐Kuka M , Liepins J , Cirule H , Gustina D , et al. Suppression of intestinal microbiota‐dependent production of pro‐atherogenic trimethylamine N‐oxide by shifting L‐carnitine microbial degradation. Life Sci 2014;117:84–92. 25301199 10.1016/j.lfs.2014.09.028

[98] Dambrova M , Skapare‐Makarova E , Konrade I , Pugovics O , Grinberga S , Tirzite D , et al. Meldonium decreases the diet‐increased plasma levels of trimethylamine N‐oxide, a metabolite associated with atherosclerosis. J Clin Pharmacol 2013;53:1095–8. 23893520 10.1002/jcph.135

[99] Tang WH , Hazen SL . The contributory role of gut microbiota in cardiovascular disease. J Clin Invest 2014;124(10):4204–11. 25271725 10.1172/JCI72331 PMC4215189

[100] Wu WK , Chen CC , Liu PY , Panyod S , Liao BY , Chen PC , et al. Identification of TMAO‐producer phenotype and host‐diet‐gut dysbiosis by carnitine challenge test in human and germ‐free mice. Gut 2019;68(8):1439–49. 30377191 10.1136/gutjnl-2018-317155 PMC6691853

[101] Yonemori KM , Lim U , Koga KR , Wilkens LR , Au D , Boushey CJ , et al. Dietary choline and betaine intakes vary in an adult multiethnic population. J Nutr 2013;143:894–9. 23616508 10.3945/jn.112.171132 PMC3652885

[102] Miller CA , Corbin KD , da Costa KA , Zhang S , Zhao X , Galanko JA , et al. Effect of egg ingestion on trimethylamine‐N‐oxide production in humans: a randomized, controlled, dose‐response study. Am J Clin Nutr 2014;100:778–86. 24944063 10.3945/ajcn.114.087692 PMC4135488

[103] Petriz BA , Castro AP , Almeida JA , Gomes CP , Fernandes GR , Kruger RH , et al. Exercise induction of gut microbiota modifications in obese, non‐obese and hypertensive rats. BMC Genomics 2014;15:511. 24952588 10.1186/1471-2164-15-511 PMC4082611

[104] Denou E , Marcinko K , Surette MG , Steinberg GR , Schertzer JD . High‐intensity exercise training increases the diversity and metabolic capacity of the mouse distal gut microbiota during diet‐induced obesity. Am J Physiol Endocrinol Metab 2016;310:E982–93. 27117007 10.1152/ajpendo.00537.2015 PMC4935139

[105] Erickson ML , Malin SK , Wang Z , Brown JM , Hazen SL , Kirwan JP . Effects of lifestyle intervention on plasma trimethylamine N‐oxide in obese adults. Nutrients 2019;11:179. 10.3390/nu11010179 PMC6356515 30654453

[106] Velasquez M , Ramezani A , Manal A , Raj D . Trimethylamine N‐oxide: the good, the bad and the unknown. Toxins 2016;8:326. 10.3390/toxins8110326 PMC5127123 27834801

[107] Hartiala J , Bennett BJ , Tang WH , Wang Z , Stewart AF , Roberts R , et al. Comparative genome‐wide association studies in mice and humans for trimethylamine N‐oxide, a proatherogenic metabolite of choline and L‐carnitine. Arterioscler Thromb Vasc Biol 2014;34:1307–13. 24675659 10.1161/ATVBAHA.114.303252 PMC4035110

[108] Shih DM , Zhu W , Schugar RC , Meng Y , Jia X , Miikeda A , et al. Genetic deficiency of flavin‐containing monooxygenase 3 (Fmo3) protects against thrombosis but has only a minor effect on plasma lipid levels‐brief report. Arterioscler Thromb Vasc Biol 2019;39:1045–54. 31070450 10.1161/ATVBAHA.119.312592 PMC6531332

[109] Andraws R , Berger JS , Brown DL . Effects of antibiotic therapy on outcomes of patients with coronary artery disease: a meta‐analysis of randomized controlled trials. JAMA 2005;293:2641–7. 15928286 10.1001/jama.293.21.2641

[110] Shokryazdan P , Faseleh Jahromi M , Navidshad B , Liang JB . Effects of prebiotics on immune system and cytokine expression. Med Microbiol Immunol 2017;206:1–9. 27704207 10.1007/s00430-016-0481-y

[111] Qiu L , Tao X , Xiong H , Yu J , Wei H . Lactobacillus plantarum ZDY04 exhibits a strain‐specific property of lowering TMAO via the modulation of gut microbiota in mice. Food Funct 2018;9:4299–309. 30039147 10.1039/c8fo00349a

[112] Jung CM , Heinze TM , Schnackenberg LK , Mullis LB , Elkins SA , Elkins CA , et al. Interaction of dietary resveratrol with animal‐associated bacteria. FEMS Microbiol Lett 2009;297:266–73. 19566680 10.1111/j.1574-6968.2009.01691.x

[113] Qiao Y , Sun J , Xia S , Tang X , Shi Y , Le G . Effects of resveratrol on gut microbiota and fat storage in a mouse model with high‐fat‐induced obesity. Food Funct 2014;5:1241–9. 24722352 10.1039/c3fo60630a

[114] Chen ML , Yi L , Zhang Y , Zhou X , Ran L , Yang J , et al. Resveratrol attenuates trimethylamine‐N‐oxide (TMAO)‐induced atherosclerosis by regulating TMAO synthesis and bile acid metabolism via remodeling of the gut microbiota. MBio 2016;7:e02210–5. 27048804 10.1128/mBio.02210-15 PMC4817264

[115] Qiu L , Yang D , Tao X , Yu J , Xiong H , Wei H . Enterobacter aerogenes ZDY01 attenuates choline‐induced trimethylamine N‐oxide levels by remodeling gut microbiota in Mice. J Microbiol Biotechnol 2017;27:1491–9. 28511293 10.4014/jmb.1703.03039

[116] Brugere JF , Borrel G , Gaci N , Tottey W , O'Toole PW , Malpuech‐Brugere C . Archaebiotics: proposed therapeutic use of archaea to prevent trimethylaminuria and cardiovascular disease. Gut Microbes 2014;5:5–10. 24247281 10.4161/gmic.26749 PMC4049937

